# Cranial morphology in the brachygnathic sheep

**DOI:** 10.1186/s12917-016-0634-7

**Published:** 2016-01-09

**Authors:** T. Eriksen, M. Ganter, O. Distl, C. Staszyk

**Affiliations:** Institute of Anatomy, University of Veterinary Medicine Hannover, Bischofsholer Damm 15, D-30173 Hannover, Germany; Clinic for Swine, Small Ruminants, Forensic Medicine and Ambulatory Service, University of Veterinary Medicine Hannover, Bischofsholer Damm 15, D-30173 Hannover, Germany; Institute for Animal Breeding and Genetics, University of Veterinary Medicine Hannover, Bünteweg 17p, D-30559 Hannover, Germany; Institute of Veterinary Anatomy, Histology and Embryology, Faculty of Veterinary Medicine, Justus-Liebig-University Giessen, Frankfurter Str. 98, D-35392 Giessen, Germany

**Keywords:** Brachygnathia inferior, Cluster analyse, Principal component analyse, Sheep, Skull

## Abstract

**Background:**

Craniofacial morphology of sheep with phenotypically observed mandibular distocclusion was analysed using the multivariate techniques principle component analysis and cluster analysis in order to test whether different types of craniofacial malformations can be distinguished.

**Results:**

The results showed 8 principal components with a variance of 82.72 % in the database. The method creates new variables then used in the Cluster analysis indicating 7 clusters with 3 different facial types: Normal, prognathia inferior and brachygnathia inferior.

**Conclusion:**

The brachygnathic facial type was mainly characterised as a shortened mandible, the upper jaw is not significantly involved. The correlations to the temporomandibular joint were shown. Molar and premolar malocclusions were revealed in two of three Clusters. Phenotypical distocclusion was not a single criterion for the affected sheep.

## Background

Dysgnathia appears as brachygnathia inferior [1–4] or brachygnathia superior [5]. Other terms used for brachygnathia inferior are overbite, undershot, parrot mouth, prognathia superior whereas brachygnathia superior is referred as underbite, overshot, prognathia inferior [1, 3, 5, 6]. Dysgnathia is a common congenital anomaly in sheep that could be reproduced in a breeding trial with East Friesian sheep [1, 7]. Due to the shortening of specific parts of the mandible, the ovine craniofacial malformation is best described as brachygnathia inferior in sheep [1, 8].

Different measurement approaches have been chosen in sheep with brachygnathia inferior. Nordby et al. [5] found a shortening of the interalveolar part of the mandible and an elongation of the rostral part of the maxilla. The measurements of premolar/molar parts were actually also considered by [5] but sheep with brachygnathia inferior did not show any deviations from standard measurements in these parameters. Measurements of the mandibular distocclusion were frequently based on the distance of the rostral point of the incisor to the dental pad [5, 9, 10]. As indicated by [8] with descriptive statistic programs, different particular parts of the mandible succumb to shortening; other craniofacial abnormalities in sheep were not identified in this study.

As found in the above cited literature (except [8]), measurements were carried out using macerated skulls. Today, due to the progressive development of x-ray technology, animals can also be assessed in vivo. As a starting point for the following study, advanced techniques used for evaluation of x-rays in orthodontics human medicine were adopted. The laterolateral x-ray can be analysed by means of cephalometric measurement points. Structures or positional relationships of skull bones, soft tissues, growth forecasts or treatment options will be shown.

According to [11], there is no uniform nomenclature or classification for the different types of dysgnathia. Even today, the starting point for diagnosing dysgnathia is the classification according to [12] based on molar teeth-occlusion. This is the same also internationally.

Angle-Class I defines normocclusion; Angle-Class II classifies the rostral placement of the maxillary first molar and is subdivided into two groups. Shortened mandible and elongated maxilla and the mandible in posterior position and the maxilla in anterior position [13]. Angle-Class III is used to classify a caudal placement of the maxillary first molar.

Additionally the different affected two Angle Classes could share in altered region (alveolar, skeletal) and changes in main directions (vertical, sagittal and transverse) [11]. Gattinger and Obwegeser [14] divided the malocclusion in the hypo- / hyperplasia of mandible and maxilla.

The projection onto affected sheep, we are trying to implement in this study. A valid normocclusion for the sheep shall be found. Additional changes of other bones of skull which are affected by malformations will be proved. Based on the current human medical knowledge, we will try to find a descriptive definition to represent the exact localization of changes by applying multivariate statistics.

## Methods

Digital radiographs of the head were taken from 101 juvenile and adult sheep including mainly East Friesian milk sheep. These were part of a breeding experiment from 2005 to 2008 conducted over four generations approved by the federal state government of Hannover, Germany (AZ: 05/ 918). An obviously affected goat with brachygnathia inferior was mated to both affected and clinically healthy sheep. The next generations have also been used for breeding and mated with each other as well as with the founder animals.

**Fig. 1:**
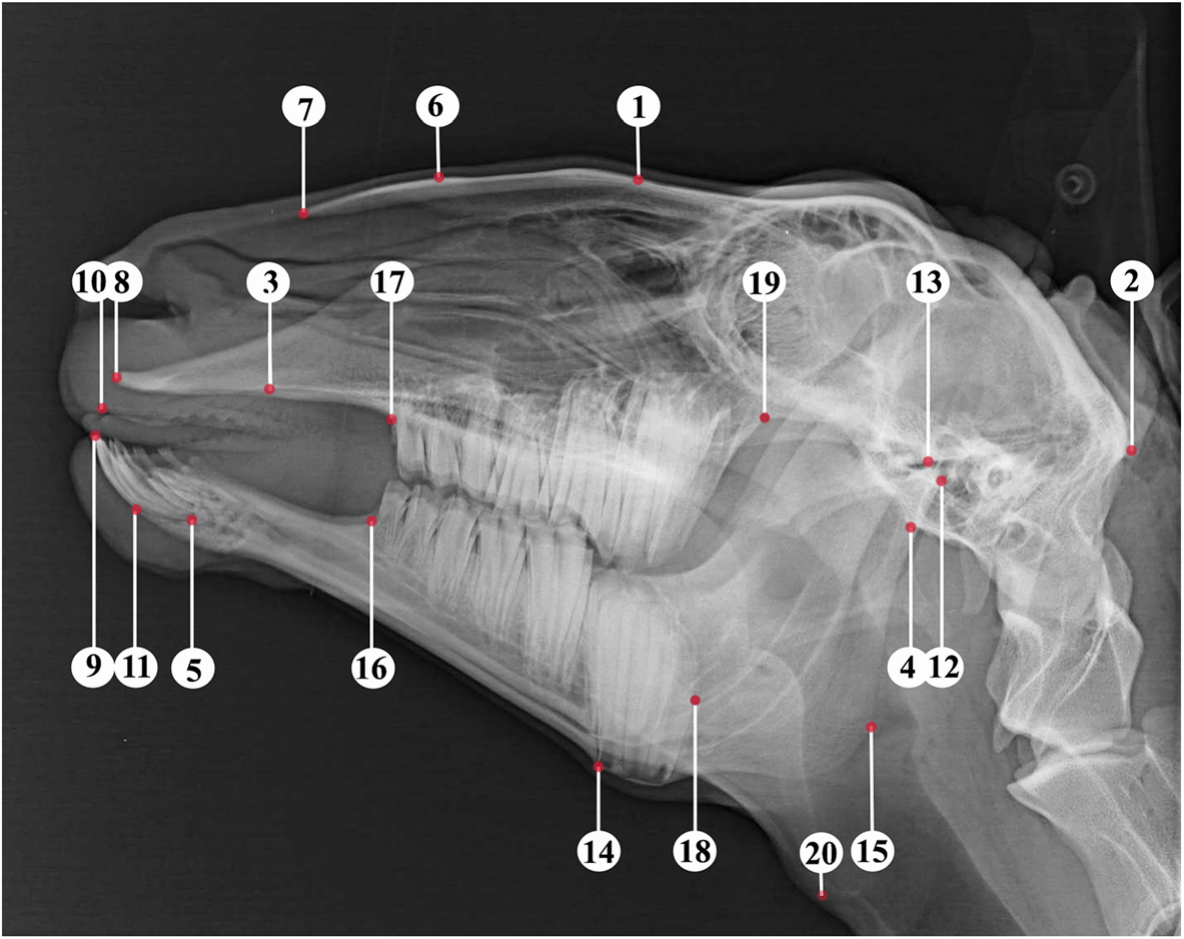
Laterolateral radiograph of the skull of a 6-year-old female sheep. *Numbers* indicate reference points ([8], figure reproduced with the permission of the publisher)

**Fig. 2:**
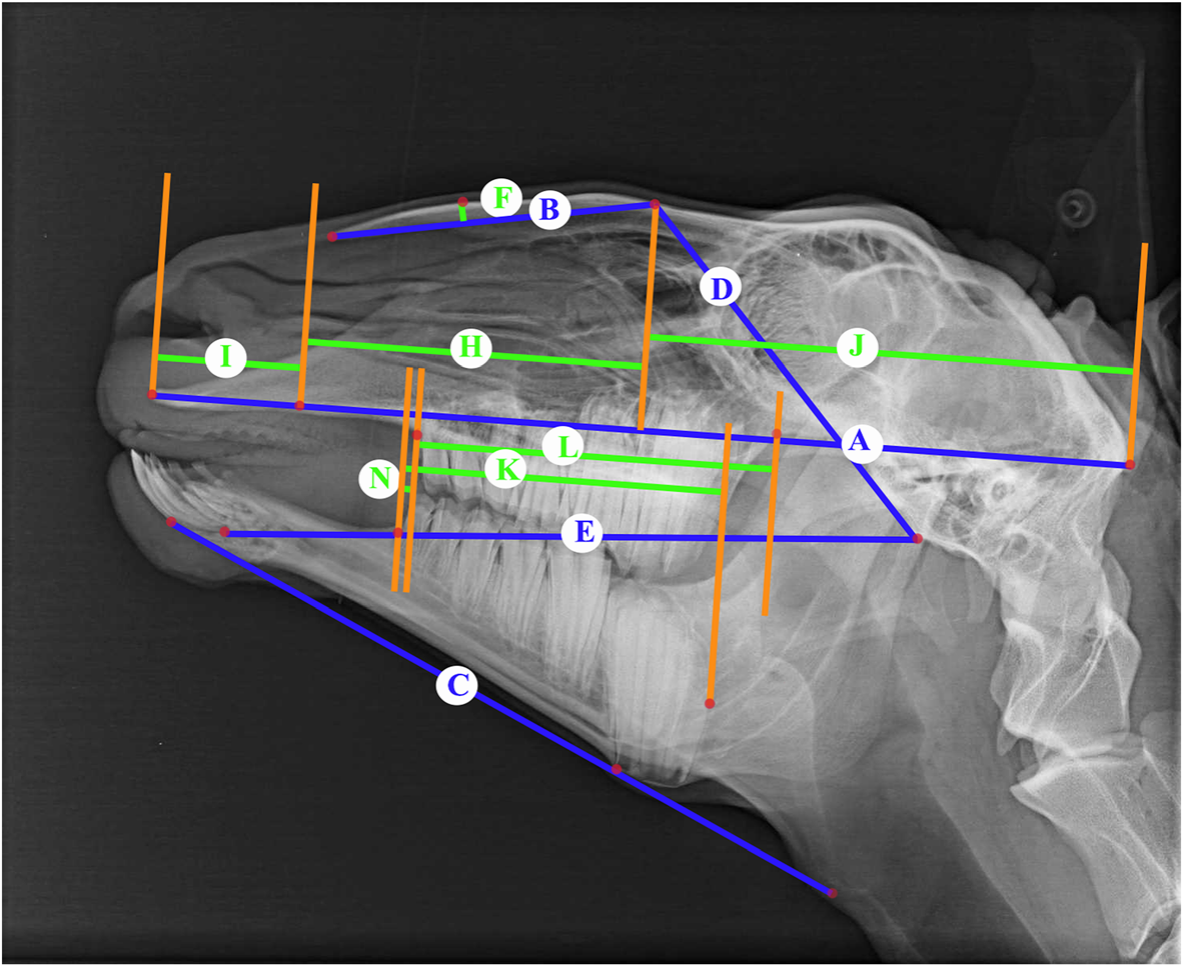
Laterolateral radiograph of the skull of a 6-year-old female sheep. Characters indicate reference lines (*blue*), perpendicular (*orange*) and measure-lines (*green*) ([8], figure reproduced with the permission of the publisher)

**Fig. 3:**
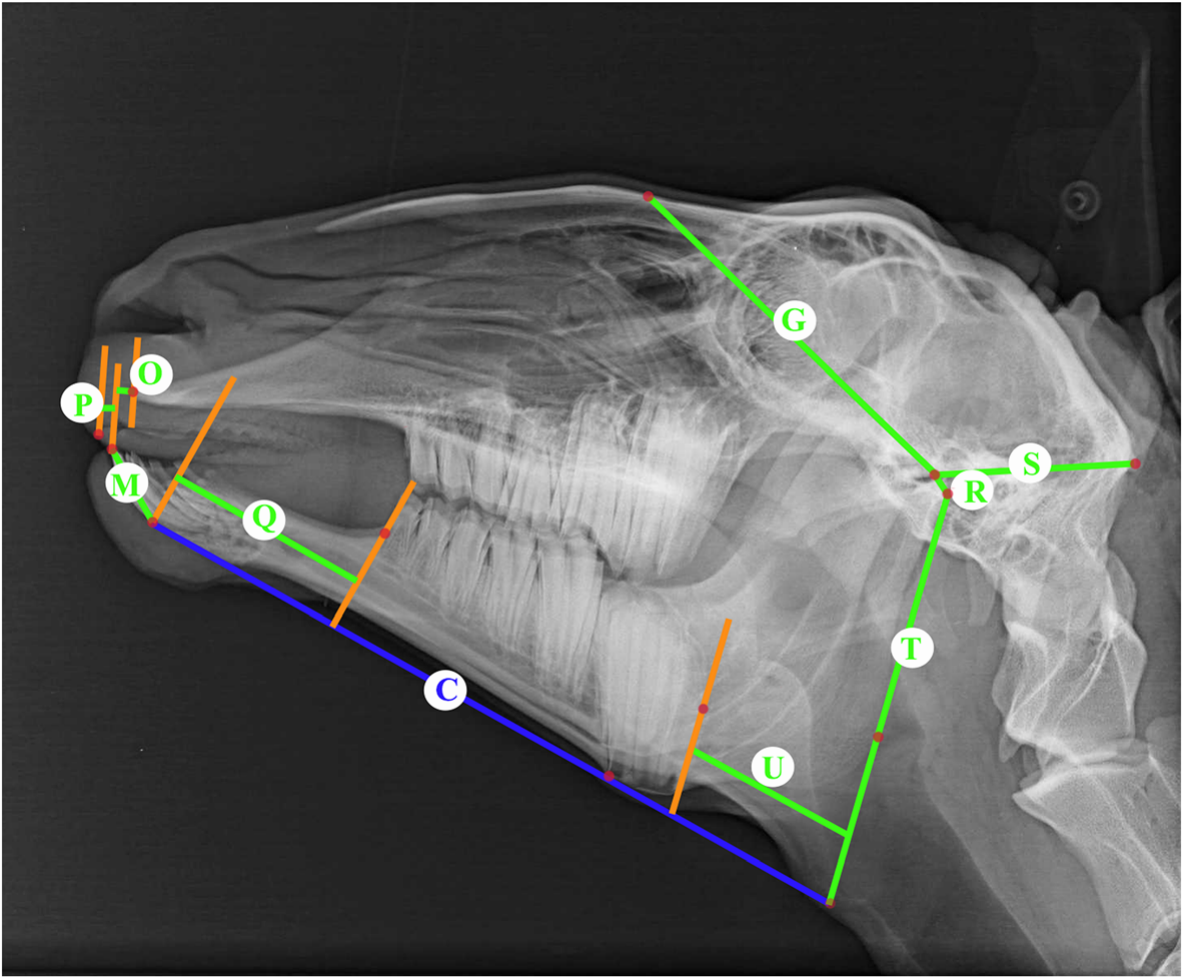
Laterolateral radiograph of the skull of a 6-year-old female sheep. Characters indicate the mandible-line (*blue*), perpendicular (*orange*) and measure-lines (*green*) ([8], figure reproduced with the permission of the publisher)

**Fig. 4:**
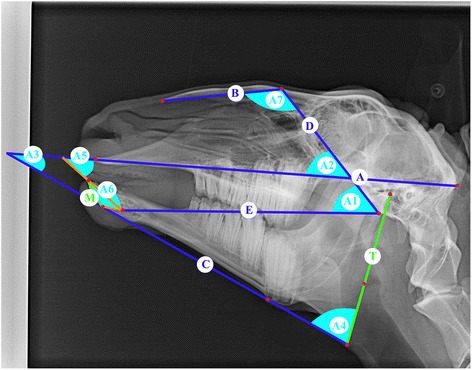
Laterolateral radiograph of the skull of a 6-year-old female sheep. Characters indicate measured angles (*turquoise*) ([8], figure reproduced with the permission of the publisher)

Latero-lateral x-rays of the skull were taken, which included cadaveric and living animals, whereas the condition of the animals showed no differences in quality. The radiographic reference points and methods from previous study were applied for our analysis [8]. The reference points are dorsal point of the frontonasal suture (1), external occipital protuberance (2), suture between maxilla and the incisive bone (3), ventral point of the sphenoid bone (4), caudal point of the alveolar process of the first incisor (5), dorsum nasi (6), rostral point of the rostral process of the nasal bone (7), rostral point of the incisive bone (8), labial border of the incisors (9), rostral point of the dental pad (10), rostral point of the mandible (11), caudo-dorsal part of the mandibular condyle (12), dorso-median point of the mandibular fossa (13), ventral point of the corpus mandibulae (14), caudal point of the ramus mandibulae (15), rostral point of the lower second premolar (16), rostral point of the upper second premolar (17), caudal point of the lower third molar (18), caudal point of the upper third molar (19), designed intercept point of the tangents from references points 14 and 15 (20) (Fig. 1 [8]). From these reference points the reference lines are derived: Baseline (A), bridge-line (B), mandible-line (C), skull-line (D), sphenoid-mandible-line (E) (Fig. 2 [8]). Sixteen measurements were determined by reference points, reference lines and perpendiculars (Figs. 3 and 4 [8]).

The measurements were standardized to a relative scale using the skull-line to minimize the effects of the different skull sizes. A generalized linear model (GLM) was used to remove the fixed effects of sex and age. The residuals were standardized onto a mean of zero and a standard deviation of 1.

Principal Component Analysis (PCA) was used to calculate eigenvalues and eigenvectors. Eigenvalues larger than one were significant. The factor scores for the individuals were calculated for new variables. The following Cluster analysis summarized the multidimensional data space.

## Results

**Fig. 5:**
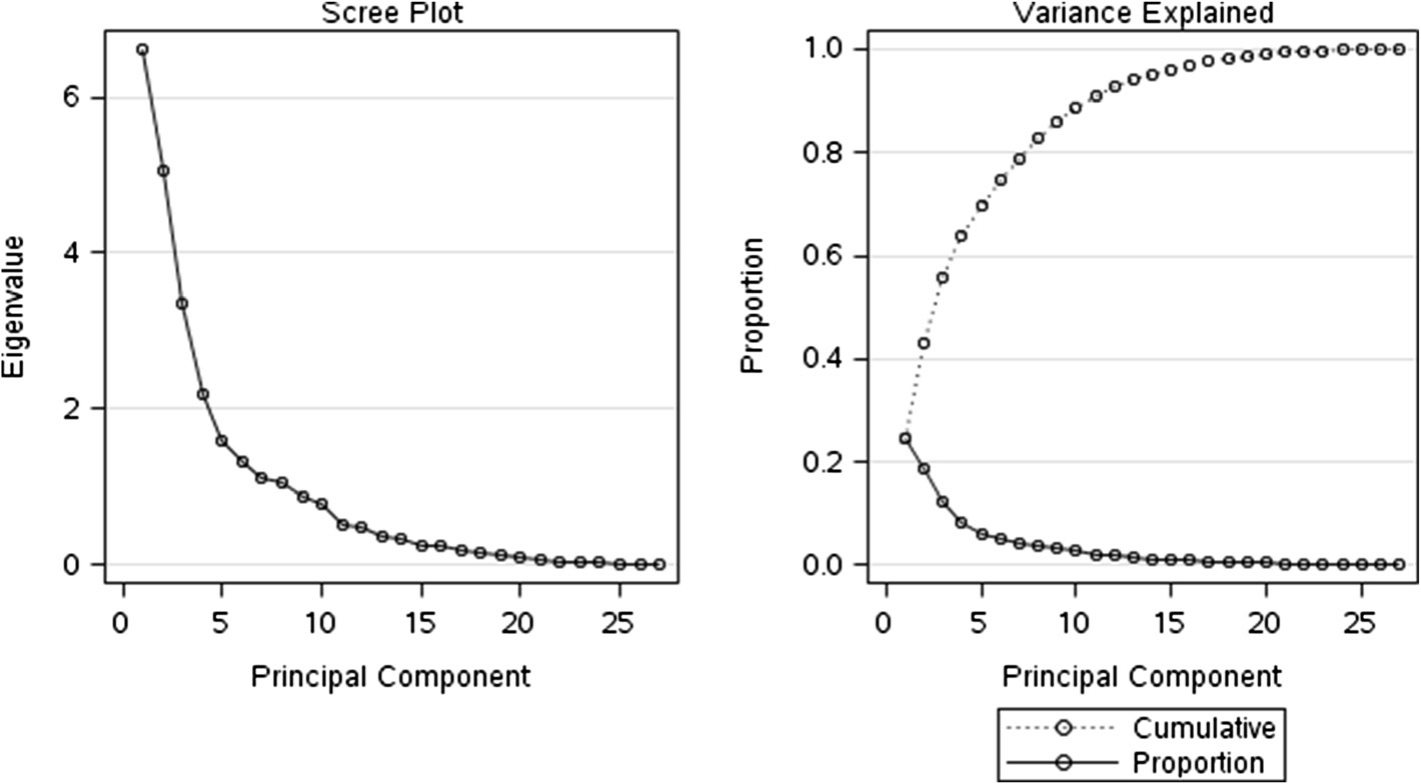
Scree plot. The variance of each PC

PCA could extract eight principal components (PC) with eigenvalues >1 using measurements normalized onto a standard normal distribution. The variance captured by these 8 PCS amounted to 82.72 % (Table 1). A scree plot graphically shows the variance of each PC (Fig. 5). The loadings of the eigenvectors indicate the proportion of variance attributable to the single measurements (Table 2).

**Table 1:** Variance of the Principal component

	Eigenvalue	Difference	Proportion	Cumulative
1	6.6145437	1.5596872	0.245	0.245
2	5.0548565	1.6915844	0.1872	0.4322
3	3.3632722	1.1787174	0.1246	0.5568
4	2.1845548	0.5926245	0.0809	0.6377
5	1.5919303	0.2637301	0.059	0.6966
6	1.3282002	0.1990405	0.0492	0.7458
7	1.1291597	0.0617238	0.0418	0.7876
8	1.0674359	0.1894865	0.0395	0.8272
9	0.8779494	0.1062893	0.0325	0.8597

**Table 2:** Loadings of the eigenvectors (standardised measurements of the skull) for principal components (Prin1- Prin8)

	Prin1	Prin2	Prin3	Prin4	Prin5	Prin6	Prin7	Prin8
b	0.260512	0.16943	0.138514	−0.31995	0.004763	−0.109674	0.024279	0.166586
a	0.210661	0.239707	0.316499	0.081616	−0.103967	−0.075809	−0.063305	−0.003094
F	0.203304	0.08517	−0.020909	−0.343357	−0.067752	−0.282682	0.040735	0.289523
g	0.048255	−0.222811	0.131433	−0.059791	−0.119593	0.206556	−0.437608	−0.053805
i	−0.059117	0.226485	0.169766	0.047072	−0.043878	0.479737	−0.020836	0.443369
h	0.279343	0.025555	0.042639	−0.311058	0.014902	−0.308813	0.032494	−0.127343
j	0.044539	0.176961	0.311619	0.401739	−0.129603	−0.138888	−0.079028	−0.192877
l	0.129432	0.184688	0.165806	−0.187116	0.313292	0.288096	0.010792	−0.141651
s	0.107072	0.211717	0.229355	0.374218	−0.072467	−0.219437	0.128797	−0.076414
e	0.177303	0.339295	−0.001585	0.085101	0.030029	0.037648	−0.000223	0.003641
k	0.083634	0.191221	0.152947	0.00207	0.532159	0.060321	0.098297	−0.249258
m	0.026714	−0.064074	0.167117	−0.132598	0.48245	−0.193502	−0.113828	0.053702
n	−0.111563	−0.12682	0.385271	−0.184432	−0.234753	−0.012267	−0.03785	−0.16482
o	0.141251	0.106741	−0.426021	0.19523	0.131828	−0.103627	−0.088336	0.006431
p	−0.1376	−0.109094	0.434017	−0.178565	−0.109052	0.100598	0.04942	0.016676
q	0.216362	0.191487	−0.082302	0.006879	−0.209547	0.034904	−0.334733	0.202341
r	−0.104137	0.249953	0.023715	0.051262	−0.164301	−0.04845	0.47971	0.280733
c	0.272892	0.17652	−0.156788	−0.019913	−0.136846	0.254788	−0.079413	−0.192924
t	0.243845	0.081729	−0.074399	−0.107708	−0.086393	0.349988	0.311518	−0.190954
u	0.206073	0.091023	−0.076007	−0.122156	−0.193091	0.050122	−0.280342	−0.226584
a1	0.302149	−0.215531	0.060091	0.036928	−0.027133	−0.030557	0.027869	0.189831
a2	0.294529	−0.226151	−0.027649	−0.010197	0.005965	0.052971	0.072626	0.224069
a3	0.17492	−0.266372	−0.023934	−0.05119	−0.033548	0.206697	0.398068	−0.076868
a4	0.226399	−0.209417	0.131739	0.203875	0.060208	−0.162003	−0.014389	0.246418
a5	0.11023	−0.124019	0.104516	0.239457	0.318966	0.229965	−0.173376	0.288112
a6	0.255131	−0.257778	0.016076	0.182813	−0.046198	0.007964	0.129697	−0.170123
a7	0.265894	−0.245075	0.093553	0.198069	−0.05404	0.032331	0.088314	−0.086675

The first PC was mainly composed of the angles. The second PC was characterised by variables describing mandible and the third PC by mandibular distocclusion. The fourth PC was mainly composed of measurements of the cranium and the fifth PC of incisor length and measurements of the pars alveolare from the check teeth. The sixth PC was characterised by measurements of the mandible and maxilla. The seventh PC was mainly composed of measurements describing the temporomandibular joint and the eighth PC of measurement of the incisive bone.

**Fig. 6:**
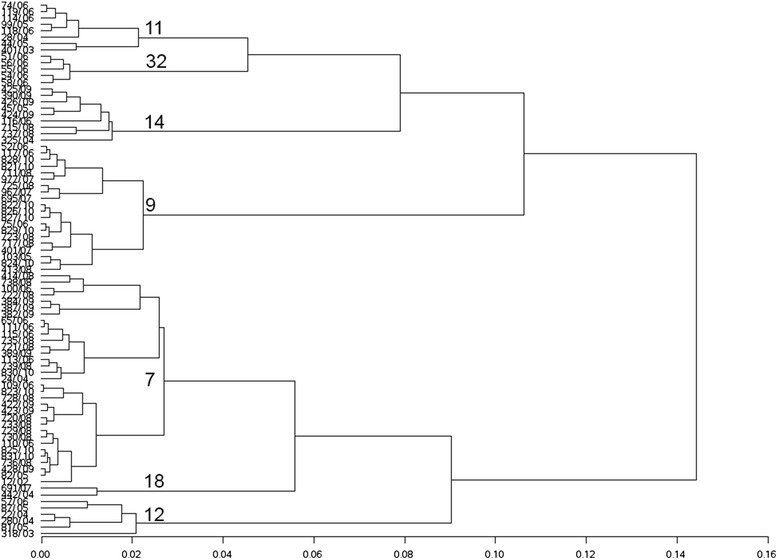
Cluster-analyse in the tree dendrogram. Name of clusters and observations. The first *row* represents each individual sheep. The remaining branches show the potential clusters

For each individual sheep the factor scores were evaluated and used as a new variable in the cluster analysis with the Ward method. The cubic clustering criterion (CCC) was used to differentiate significant from non-significant clusters. In total, seven significant clusters of the craniofacial malformation could be distinguished (Fig. 6). The largest cluster (cluster no. 7) contained 33 animals (40 %) and the smallest cluster (cluster no. 18) consisted of 2 animals (Table 3).

**Table 3:** Observation (*n*) frequency of different cluster types

Cluster (Type)	Frequency (*n*)	Percent (%)
7	33	39.76
9	20	24.1
11	8	9.64
12	6	7.23
14	9	10.84
18	2	2.41
32	5	6.02

The common characteristic of cluster 14 with nine animals is an elongation of the incisive bone (I), the cerebral cranium (J) and the shortening of the maxilla (H). The temporomandibular joint (G) was in caudal position. Cluster 32 differs mainly in shortening of the incisive bone (I) and the cerebral cranium (J). Cluster 11 shows an elongation of the maxilla (H) and the nose (B and F). The temporomandibular joint (G) was in rostral position. The measure-lines C and E (mandible-line) were reduced and the measure-line M (incisor length) was elongated like the rest in the upper types. Only clusters 32 and 14 were phenotypically classified as brachygnathic sheep.

**Fig. 7:**
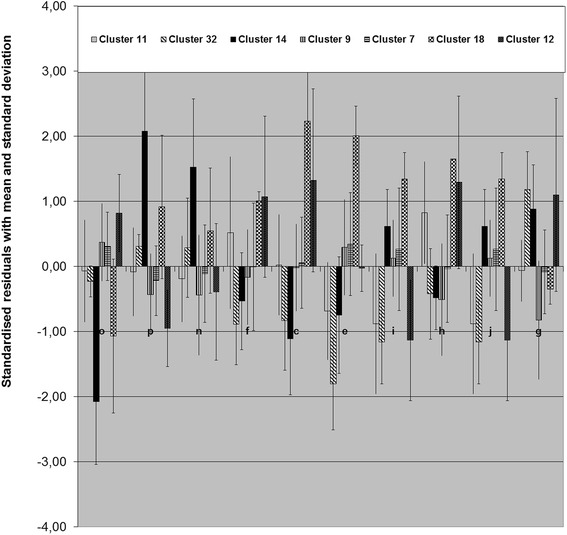
Means and standard deviations for all animals in the certain clusters. Positive and negative standard deviation (end of the whiskers) from the mean (*top* of the box) of the population

Clusters 9, 7 and 12 were classified as normal or prognathia inferior and were identified by the measure-lines p, o and n. The measure-lines C and E were elongated. The measure-lines B and F, affecting the nose, were in median or in elongation. Differences were identified in measure-lines which concerned the upper jaw. Cluster 9 was characterised by an elongated incisive bone (I) and baseline (A) and shortening of the maxilla (H) and cerebral cranium (J). Cluster 7 differs from cluster 9 and 12 with an elongation of the cerebral cranium (J). Cluster 12 is characterised by an elongated maxilla (H) and shortening of the incisive bone (I) and cerebral cranium (J). Only in Cluster 12, the temporomandibular joint (G) was in caudal position (Fig. 7).

## Discussion

Facial types could be defined in our analysis that could applied for the description of dysgnathia. The cluster 32, 11 and 14 were classified as affected sheep with shortening of the mandible. The discrepancy between the position of the mandibular incisor and the dental pad [1, 10] was not a single criterion for an affected sheep.

The craniofacial malformation should be defined as brachygnathia inferior because the lower jaw is in all cases shortened [5, 8]. The upper jaw is involved but of only limited informative value for the definition of the brachygnathia inferior. Molar and premolar malocclusions of different types were shown (measure-line N). In comparison to the horse, it was interesting that in sheep the malformation was only restricted to the rostral part of the mandible. The molar and premolar occlusion was commonly not affected [15].

The phenotypical classification was not in accordance with cluster 11, even though the shortening of the mandible exists. The temporomandibular joint (measure-line G) was in rostral position and the mandibular incisor (measure-line M) was elongated, so the length of the mandible was apparently corrected. In our study there were statistically more affected sheep than from a clinical point of view shown. The temporomandibular joint was in correlation with the mandible in brachygnathic sheep.

The other 3 facial types were classified as prognathia inferior or normal. In the cluster 9 and 7, the maxilla was elongated with a more rostral location from the temporomandibular joint. In contrast, in cluster 12, the temporomandibular joint was in the caudal position. What remains for discussion is the influence of the temporomandibular joint to the dysgnathia.

In human medicine, the focus on the temporomandibular joint was in correlation to the mandible bone [16, 17]. There were examinations with focus on the temporomandibular joint and the dysgnathia. As a result, in Angle Class II the fossa mandibularis shifts in the caudal position [18, 19]. In order to achieve more detailed information about the correlation of the temporomandibular joint and the brachygnathia inferior a three-dimensional view like the computer tomography (CT) is vital. Based on cross-sectional images the skull can be reconstructed with special software programs.

## Conclusions

The brachygnathia inferior is characterised as a significant shortened mandible. The distance between the position of the mandibular incisor and the dental pad is not a single criterion.

## Abbreviations

CCC: cubic clustering criterion
CT: computer tomography
Fig.: figure
GLM: generalized linear model
No.: number
PC: principal component
PCA: principal component analyse
